# Complete chloroplast genome of *Atriplex taltalensis* (Amaranthaceae), an endangered endemic species from the Chilean Atacama Desert

**DOI:** 10.3389/fpls.2026.1975705

**Published:** 2026-10-26

**Authors:** Francisca Peña, Antü Salas-Tarque-Huanca, Luciano Univaso, Celián Román-Figueroa, Manuel Paneque

**Affiliations:** 1 Agroenergía Ingeniería Genética S.A. Inc., Santiago, Chile; 2 School of Environmental Sciences and Biotechnology, Faculty of Science, University of Chile, Santiago, Chile; 3 Bionostra Chile Research Foundation, Santiago, Chile; 4 Department of Environmental Sciences and Natural Resources, Faculty of Agricultural Sciences, University of Chile, Santiago, Chile

**Keywords:** Atriplex taltalensis, chloroplast genome, comparative plastomics, conservation genomics, molecular markers, nucleotide diversity, phylogenomics

## Abstract

**Background:**

*Atriplex taltalensis* is an endangered halophytic species endemic to the Chilean Atacama Desert, for which genomic resources are currently lacking. Complete chloroplast genomes provide valuable resources for comparative genomics, phylogenetics, and conservation genetics because of their conserved structure and informative evolutionary patterns. This study aimed to assemble and characterize the first complete chloroplast genome of *A. taltalensis* and compare it with representative *Atriplex* plastomes.

**Results:**

The complete chloroplast genome of *A. taltalensis* was a circular molecule of 150, 919 bp with the typical quadripartite structure, comprising a large single-copy region, a small single-copy region, and two inverted repeats. The plastome encoded 130 genes, including 85 protein-coding genes, 37 tRNAs, and 8 rRNAs, with an overall GC content of 37.29%. Comparative analyses of 15 *Atriplex* plastomes revealed high conservation of genome organization and gene content, together with high sequence similarity, whereas nucleotide variation was concentrated in a limited number of intergenic regions and a few coding genes. Hypervariable loci, simple sequence repeats, and long repeats were identified as potential candidates for informative molecular markers. Most protein-coding genes showed Ka/Ks ratios consistent with strong functional constraint, while a limited number of genes exhibited elevated pairwise Ka/Ks ratios in specific comparisons. Phylogenomic reconstruction based on complete plastomes recovered well-supported plastome relationships within *Atriplex*, supporting their utility for resolving relationships among closely related taxa.

**Conclusions:**

This study provides the first complete chloroplast genome sequence of *A. taltalensis* and establishes a valuable genomic resource for comparative genomics, phylogenomics, and conservation genetics.

## 1 Introduction

The genus *Atriplex* L. (Amaranthaceae) comprises approximately 250–300 species distributed globally across arid and semi-arid landscapes (Román-Figueroa et al., 2019). These halophytic species play a fundamental role in the resilience of desert and coastal ecosystems and possess remarkable adaptive mechanisms that enable them to tolerate high salinity, water scarcity, and thermal stress (Žerdoner Čalasan et al., 2022). The genus has become a focal point for studies in ecology, environmental restoration, and evolutionary responses to climate change (Benzarti et al., 2013; Fernández et al., 2016; Shuyskaya et al., 2019).

In Chile, the genus is represented by 29 species, including 14 endemic taxa (Román-Figueroa et al., 2019). Taxonomic resolution within *Atriplex* has historically been challenging, owing to substantial phenotypic plasticity, morphological convergence, and widespread hybridization, which often obscure species boundaries (Žerdoner Čalasan et al., 2022). In this context, complete chloroplast genomes provide a valuable source of phylogenetically informative characters because of their conserved genomic structure, predominantly uniparental inheritance, and relatively low recombination rates, thereby improving the resolution of evolutionary relationships among closely related taxa (Rogalski et al., 2015; Ruhlman and Jansen, 2021; Yang et al., 2022a). These characteristics make plastome sequencing a particularly suitable approach for clarifying systematic relationships and generating genomic resources for poorly studied species within the genus.

Although genomic resources for *Atriplex* have increased in recent years, published plastome studies remain scarce. To date, complete chloroplast genomes have been comprehensively characterized for only a few species, including *A. centralasiatica* (Zhang et al., 2019), *A. gmelinii* (Park et al., 2022) and *A. patens* and *A. prostrata* (Wei et al., 2023). Although additional plastome sequences are currently available in public repositories, many have not yet been included in comparative genomic studies, leaving substantial gaps in our understanding of plastome evolution across the genus.

Among the Chilean endemic species, *Atriplex taltalensis* I.M. Johnst. represents a critical component of the halophytic biodiversity of the Chilean coastal desert, particularly in the Taltal area of the Antofagasta Region (Muñoz-Schick et al., 2001; Muñoz-Alcayaga et al., 2023). In the context of climate change and habitat fragmentation, complete chloroplast genome sequencing has become a valuable tool for evolutionary studies and conservation genetics because chloroplast genomes have a conserved genomic structure, predominantly uniparental inheritance, low recombination rates, and suitability for molecular marker development*. A. taltalensis* has been officially classified as endangered because it is restricted to fragile and fragmented habitats that are increasingly threatened by anthropogenic pressures and climate change (MMA (Ministerio del Medio Ambiente), 2025). Despite its ecological importance and urgent conservation needs, genomic resources remain limited, restricting our ability to monitor natural populations and better understand the evolutionary processes underlying the species.

To date, no complete chloroplast genome of *A. taltalensis* has been reported in the published literature. The generation of a reference plastome would provide a valuable genomic resource for the development of molecular markers aimed at assessing genetic diversity, identifying conservation units, and supporting long-term conservation and management strategies. Consequently, the absence of a reference plastome has limited comparative genomic studies, the identification of informative molecular markers, and broader evolutionary, phylogeographic, and conservation research on this endangered endemic species.

To address this knowledge gap, we assembled and annotated the first complete chloroplast genome of *A. taltalensis*. We further conducted a comparative plastome analysis of representative *Atriplex* species to investigate genome organization, inverted repeat dynamics, repetitive sequences, nucleotide diversity, and phylogenetic relationships. The genomic resources generated in this study provide an important foundation for future evolutionary, phylogeographic, and conservation studies of *Atriplex* species inhabiting arid environments.

## 2 Materials and methods

### 2.1 Plant material and DNA extraction

Young leaves of *A. taltalensis* I.M. Johnst. were collected from the locality of Quebrada Cascabeles, commune of Taltal, Antofagasta Region, Chile (25°28’24.0”S, 70°24’59.0”W), from the same locality and sector previously documented for this species by Muñoz-Alcayaga et al. (2023). The plant material was transported under refrigerated conditions to the laboratory for processing. For comparative analyses, samples of *A. canescens*, *A. nummularia*, *A. atacamensis*, and *A. undulata*, obtained from the germplasm bank of Fundación Bionostra Chile Research, were also used. For each newly sampled species, leaf material from five individuals was pooled before DNA extraction and sequencing. Therefore, each newly generated accession represents one pooled sample derived from five individuals rather than five independently sequenced individuals.

Additionally, publicly available sequence data for *A. atacamensis* (SRX6414171) and *A. deserticola* (SRX6407378) were obtained from the Sequence Read Archive (NCBI) (Correa et al., 2019). Complete chloroplast genomes of additional *Atriplex* species available in GenBank were included for comparative genomic analyses. To infer phylogenetic relationships, complete chloroplast genomes of representative species from the genera *Beta*, *Chenopodium*, *Dysphania*, *Oxybasis*, and *Limonium* were incorporated as outgroups. All chloroplast genomes used in this study, together with their accession numbers and sources, are listed in **Table 1**.

**Table 1:** Chloroplast genomes included in this study.

Species name	NCBI Code	Code	Specie name	NCBI Code	Code
*A. centralasiatica*	NC_045304.1	ACA-1	*Beta vulgaris subsp. maritima*	NC_059015.1	BVSM-1
*A. patens*	ON149850.1	AP-1	*Beta vulgaris subsp. vulgaris*	NC_059012.1	BVSV-1
*A. sibirica*	ON149852.1	AS-1	*Chenopodium album*	NC_034950.1	CHA-1
*A. prostrata*	NC_084791.1	APR-1	*Chenopodium quinoa*	NC_034949.1	CHQ-1
*A. fera*	ON149857.1	AF-1	*Dysphania ambrosioides*	NC_041201.1	DA-1
*A. gmelinii*	NC_059062.1	AG-1	*Dysphania botrys*	NC_042166.1	DB-1
*A. patens*	NC_085146.1	AP-2	*Dysphania pumilio*	NC_041159.1	DP-1
*A. canescens*	NC_082104.1	AC-1	*Limonium franchetii*	NC_085143.1	LF-1
*A. deserticola*	SRX6407378	AD-1	*Oxybasis glauca*	NC_047226.1	OG-1
*A. atacamensis*	SRX6414171	AA-1			
*A. atacamensis*		AA-2			
*A. canescens*		AC-2			
*A. nummularia*		AN-1			
*A. taltalensis*		AT-1			
*A. undulata*		AU-1			

The table lists all *Atriplex* plastomes analyzed, including newly assembled genomes, publicly available sequences retrieved from GenBank and the NCBI Sequence Read Archive (SRA), and the outgroup species used for phylogenetic reconstruction. For each accession, the species name, sample code, source, and corresponding database accession number are indicated.

### 2.2 Chloroplast isolation, DNA extraction, and quality assessment

Chloroplast DNA was obtained using a chloroplast enrichment protocol followed by extraction with a modified cetyltrimethylammonium bromide method. Chloroplast isolation was performed by differential centrifugation as previously described (Kahlau et al., 2006; Yu et al., 2024). Subsequently, DNA was purified by phenol–chloroform extraction and isopropanol precipitation. The complete protocols are described in **Supplementary Materials S1** and **S2**.

DNA concentration was determined by fluorometry using a Qubit 4 instrument (Invitrogen) and the Qubit dsDNA Broad Range Assay kit (ThermoFisher Scientific). Purity was assessed by spectrophotometry (Epoch 2; BioTek), with A260/A280 ratios between 1.8 and 2.1 considered acceptable. DNA integrity was verified by amplifying the plastid genes *matK* and *rbcL* and the 18S rRNA marker by polymerase chain reaction (PCR), followed by electrophoresis on a 1% agarose gel. All samples meeting the quality criteria were used for sequencing library construction. Primer sequences and PCR conditions are provided in **Supplementary Material S3**.

### 2.3 DNA sequencing

Samples that met the concentration, purity, and integrity criteria were sent to TCL Group for library preparation and next-generation sequencing. Libraries were constructed using an MGIEasy Fast FS Library Prep Set V2.0 kit (MGI), and sequencing was performed on a DNBSEQ-G400 platform (MGI) using 150-bp paired-end sequencing (PE150). The resulting reads were delivered in FASTQ format (**Supplementary Material S4**).

### 2.4 Chloroplast genome assembly and annotation

The quality of raw reads was assessed using FastQC v.0.11.9 before and after filtering (Andrews, 2010). Adapter removal and quality-based trimming were performed using Atria v.3.0.1 with default parameters (Chuan et al., 2021).

Chloroplast genomes were assembled and characterized using GetOrganelle v.1.7.7 and the embryophyte plastome-specific mode (embplant_pt) (Jin et al., 2020). k-mer values of 21, 45, 65, 85, and 105 were used, with a maximum of 15 extension iterations. The plastome of *A. centralasiatica* (NC_045304) was used as the initial reference. The structure and circularity of the assemblies were verified using Bandage v.0.8.1 (Wick et al., 2015). Additionally, the original reads were mapped back to the assembled plastomes to assess coverage depth (103×–445×) and identify potential structural chimeras at the borders of repetitive regions.

Gene annotation was performed independently using CpGAVAS2 and PGA (Qu et al., 2019; Shi et al., 2019). Subsequently, the resulting annotations were compared and refined using ChloroAnno (Hua, 2020), yielding a consensus annotation for each genome. Manual review of gene boundaries and coding and non-coding regions was performed using Apollo (Dunn et al., 2019). For comparative gene-content assessment, gene names were standardized across annotations, and duplicated features located within the inverted repeat regions were distinguished from unique gene identities. Total gene-copy counts included duplicated IR copies, whereas comparisons of gene-content conservation were based on unique gene identities. Annotations were also examined for pseudogenes, anomalous features, and genes apparently absent from individual plastomes to distinguish potential biological differences from discrepancies in annotation.

### 2.5 Comparative characterization of the plastome

To evaluate the structural conservation of plastomes in the genus *Atriplex*, eight complete chloroplast genomes available in GenBank were analyzed along with those assembled in this study. The sequences were reoriented using heuristic for Cyclic Edit Distance (hCED) and visually verified in AliView to ensure consistent orientation (Larsson, 2014; Ayad et al., 2017).

The organization of the chloroplast genome and the positions of the large single-copy (LSC), small single-copy (SSC), and inverted repeat (IR) regions were visualized using Chloroplot (Zheng et al., 2020), whereas expansions and contractions of the IR regions were examined using CPJSdraw (Li et al., 2023).

Microsatellites (SSRs) were identified with MISA software (Beier et al., 2017). MISA was configured to detect mononucleotide repeats with ≥10 repeat units, dinucleotide repeats with ≥5 repeat units, trinucleotide repeats with ≥4 repeat units, and tetra-, penta-, and hexanucleotide repeats with ≥3 repeat units. The maximum interruption distance between two SSRs was set at 100 bp, and GFF output was enabled. To characterize the genomic distribution of SSRs, the MISA-generated coordinates were compared with the corresponding GenBank annotations of each plastome. SSRs overlapping annotated coding sequences (CDSs) were classified as CDS-associated, whereas SSRs located outside annotated coding sequences and other genic features were classified as intergenic spacer (IGS) SSRs. SSRs overlapping tRNA, rRNA, or other annotated genic features, but not CDSs were retained as a separate “other genic” category. This classification was performed for all 15 *Atriplex* plastomes to quantify SSR abundance according to genomic context. Long repeats were identified with REPuter (Kurtz et al., 2001), using a Hamming distance of 3, a minimum repeat length of 30 bp, and a maximum of 5, 000 computed repeats.

Genomic differences among plastomes were assessed using MUMmer4 v3.1 to identify single-nucleotide polymorphisms (SNPs) and insertions/deletions (InDels) (Marçais et al., 2018). MUMmer4 was run using nucmer followed by dnadiff with default algorithmic parameters; the reference plastome was compared independently against each analyzed plastome. Global sequence conservation was examined using mVISTA with the Shuffle-LAGAN algorithm (Brudno et al., 2003; Frazer et al., 2004).

The results generated by the different tools were integrated using an automated Python workflow developed by the authors (P1-GC). The workflow was implemented in Python 3.13.13 and uses Biopython, pandas, NumPy, scikit-allel, matplotlib, seaborn, CairoSVG, and ReportLab for sequence handling, data integration, statistical processing, visualization, and report generation. The pipeline includes automated modules for input validation and standardization of FASTA, GenBank, and GFF3 files; genome renaming and organization; plastome region identification and normalization; visualization of inverted-repeat junctions; detection and annotation of SNPs and InDels; phylogenetic analysis; codon usage analysis; and Ka/Ks estimation. Outputs generated by the different analytical steps are automatically organized into standardized tables, graphical summaries, and a final execution report, facilitating consistency and traceability across comparative plastome analyses. The source code is not currently publicly available but can be provided by the authors upon reasonable request.

The custom P1-GC workflow was used to integrate outputs from the individual analytical tools and automate downstream processing, tabulation, and visualization. For reproducibility, the parameters used for the principal analyses are provided in the corresponding methodological subsections and configuration files. The workflow version used for the present study represents an intermediate development version and was not intended to constitute a finalized software release.

### 2.6 Phylogenetic analysis

Complete plastome sequences were aligned using MAFFT v.7.490 (Katoh and Standley, 2013). Outgroups included species belonging to the genera *Chenopodium*, *Dysphania*, *Beta*, *Oxybasis* and *Limonium*, selected from sequences available in GenBank (**Table 1**).

The optimal evolutionary model was selected using ModelTest-NG v.0.1.7 (Darriba et al., 2020), based on the Bayesian Information Criterion (BIC). The best-supported model identified by ModelTest complete plastomes genomes-NG was subsequently used as the substitution model in IQ-TREE v.2.4.0 (Minh et al., 2020) for maximum-likelihood phylogenetic reconstruction. Node support was assessed using 5, 000 ultrafast bootstrap (UFBoot) replicates and 1, 000 SH-aLRT replicates. The resulting trees were visualized and edited using the ggtree library in R (Yu et al., 2016). To evaluate the phylogenetic signal of the hypervariable regions identified by nucleotide-diversity analyses, maximum-likelihood phylogenies were reconstructed for selected individual loci and concatenated datasets. Individual analyses included *ycf1*, *rpoC2*, *rpl32*, *matK*, and *rps15*, while concatenated datasets comprised selected hypervariable coding and intergenic regions, as well as the complete set of hypervariable loci. Alignments generated for these regions were analyzed using IQ-TREE under the best-fit evolutionary model, with 5, 000 UFBoot and 1, 000 SH-aLRT replicates. The resulting topologies were compared with the complete-plastome phylogeny based on the 15 *Atriplex* accessions. For this comparison, the outgroup taxa were removed from the reference tree, and topological concordance was assessed by comparing the internal bipartitions recovered in each tree.

### 2.7 Analysis of nucleotide diversity (π) and molecular evolution

Nucleotide diversity (π) was assessed across the 15 aligned *Atriplex* plastomes, excluding outgroup sequences. A custom Python script implemented a sliding-window analysis using a 100-bp window and a 25-bp step size. For each window, segregating sites (S) were identified after excluding alignment gaps (“-”) and ambiguous nucleotides (“N”) from the analysis. The mean number of pairwise nucleotide differences (K) was calculated across all pairwise comparisons, and nucleotide diversity was estimated as π = K/L_e_, where L_e_ represents the number of effective sites with more than one valid nucleotide observation. Watterson’s θ was calculated as S/(a_1_L_e_), where a_1_ is the standard harmonic-number term corresponding to the sample size. Windows with no effective sites were assigned missing values. Relative synonymous codon usage (RSCU) values were calculated using RSCUcaller (Maździarz et al., 2025). The resulting RSCU matrix was subsequently visualized using the RSCU-Plot web server (Hejazi et al., 2025; Diani Gohar and Soorni, 2025; Akrami et al., 2025; Soorni and Golchini, 2025), to generate standardized heatmap representations of codon usage patterns across the analyzed plastomes. Synonymous (Ks) and nonsynonymous (Ka) substitution rates were estimated for protein-coding genes shared across the analyzed *Atriplex* plastomes. Coding sequences (CDSs) were extracted from the annotated GenBank files, and sequences whose lengths were not multiples of three were excluded. The CDSs were translated with the plastid genetic code (translation **Table 1**1), and the resulting protein sequences were aligned using MAFFT with the --auto option. The protein alignments were subsequently back-translated to codon alignments using PAL2NAL and generated in PAML format. Ka and Ks values were then estimated using KaKs_Calculator v3.0 (Zhang, 2022) with the MA method and genetic code 11. Comparisons were performed between each analyzed plastome and the selected reference plastome. To minimize instability in estimates associated with very low synonymous substitution rates, comparisons with Ks < 0.001 were excluded from downstream interpretation. Additionally, Ka/Ks values >10 were treated as unreliable and excluded from summary statistics. Ka/Ks ratios >1 were therefore interpreted as elevated pairwise ratios rather than as definitive evidence of positive selection.

## 3 Results

### 3.1 Assembly and characterization of the chloroplast genome

The complete chloroplast genome of *A. taltalensis* was assembled as a circular molecule of 150, 919 bp with the quadripartite-structure characteristic of angiosperm plastomes, comprising one LSC region, one SSC region and two IR (IRa and IRb) regions (**Figure 1**).

**Figure 1:**
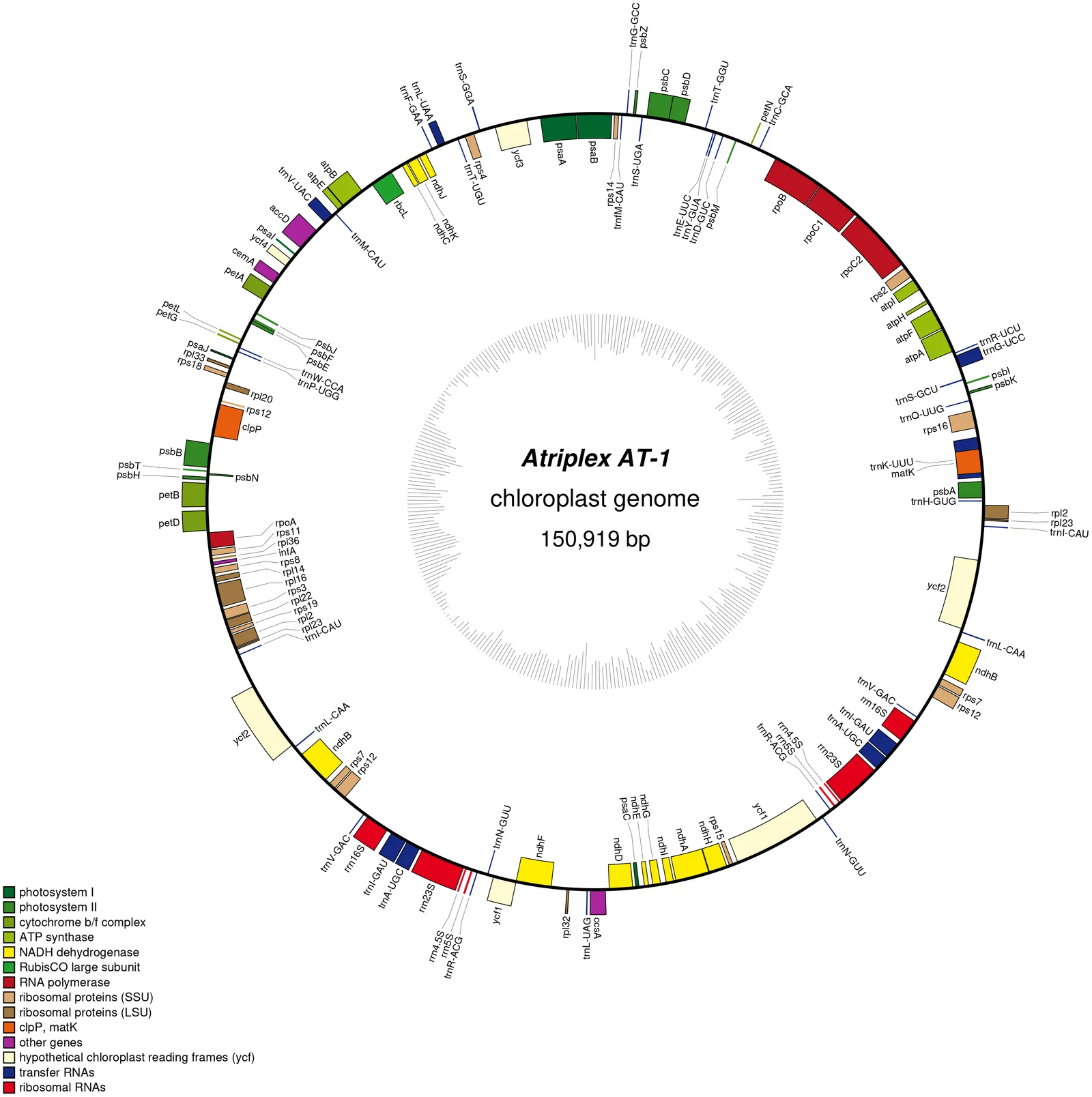
Circular map of the chloroplast genome of *A. taltalensis*. Genes located outside the circle are transcribed clockwise, and those inside are transcribed counterclockwise. Genes are colored according to their functional category. The inner circle represents the GC content throughout the genome. LSC, SSC, and IR regions are indicated on the map. Maps for the other analyzed species are presented in **Supplementary Figure S1**.

The plastome contained an LSC region of 83, 698 bp, an SSC region of 18, 073 bp, and two IR regions of 24, 574 bp each, with an overall GC content of 37.29% (**Table 2**). A total of 130 genes were identified, comprising 85 protein-coding, 37 tRNA genes, and 8 rRNA genes.

**Table 2:** General characteristics of the chloroplast genomes of *Atriplex* species analyzed in this study.

Specie of *Atriplex*	Genome size and regions (bp)				GC (%)	Genes count		
	Genome size	LSC	SSC	IR		CDS	tRNA	rRNA
ACA-1	152237	83720	18095	25209	37, 27	85	37	8
AP-1	152221	83713	18096	25204	37, 28	85	37	8
AS-1	152220	83710	18088	25209	37, 27	85	37	8
APR-1	152215	83886	17953	25186	37, 29	85	37	8
AF-1	152203	83702	18089	25204	37, 28	85	37	8
AG-1	151852	83715	17811	25161	37, 29	85	37	8
AP-2	151850	83713	17811	25161	37, 29	85	37	8
AC-1	150664	83575	18045	24520	37, 26	86	37	8
AD-1	150439	83270	18111	24529	37, 33	85	37	8
AA-1	150436	83272	18106	24529	37, 33	85	37	8
AA-2	150570	83428	18084	24529	37, 29	84	36	8
AC-2	150539	83437	18042	24530	37, 31	85	37	8
AN-1	152366	83869	18095	25201	37, 32	85	37	8
AT-1	150919	83698	18073	24574	37, 28	85	37	8
AU-1	150702	83543	18083	24538	37, 28	85	37	8

The total plastome size, length of the LSC, SSC, and IR regions, GC content (%), total number of genes, and number of protein-coding (CDS), tRNA, and rRNA genes identified in each species are presented.

The observed gene organization was consistent with that of other *Atriplex* species, with the gene order and orientation characteristic of Amaranthaceae plastomes retained. The circular genomic map showed the distribution of functional genes, transcriptional orientation, and arrangement of the LSC, SSC, and IR regions (**Figure 1**).

The newly generated plastomes showed 100% genome coverage, with sequencing depths ranging between approximately 103×–445×, supporting high quality of the assemblies (**Supplementary Table S1**).

Additionally, the RSCU of protein-coding genes was evaluated. Codon usage patterns were highly conserved among the analyzed *Atriplex* plastomes, with a clear preference for synonymous codons ending in adenine (A) or uracil (U), consistent with the relatively AT-rich composition of the plastomes (**Supplementary Figure S2**; **Supplementary Table S2**).

Overall, the assembled chloroplast genome of *A. taltalensis* exhibited the canonical quadripartite organization, gene content, and genome architecture characteristic of *Atriplex* and other angiosperm plastomes, supporting the high quality of the assembly and its suitability for subsequent comparative genomic analyses.

### 3.2 Structural and sequence conservation of chloroplast genomes

Comparison of the *Atriplex* plastomes revealed a high degree of conservation in their structural organization (**Table 2**). Genome size ranged from 150, 436 bp in *A. atacamensis* (AA-1) to 152, 366 bp in *A. nummularia* (AN-1), and the GC content remained virtually constant across all species (37.26%–37.33%). Furthermore, overall gene organization and plastome architecture were highly conserved across all analyzed plastomes, although minor differences in the numbers of annotated CDS and tRNA features were observed. Re-examination of the GenBank annotations indicated that these differences were primarily associated with annotation and gene-counting criteria rather than extensive differences in gene content.

To standardize gene-content comparisons, GenBank annotations were re-examined using common counting criteria that distinguished between total genomic gene copies and unique gene identities. Genes occurring in the two IR copies were counted as two genomic copies when reporting total gene counts, whereas gene-content conservation was assessed based on unique gene identities. This re-analysis identified several annotation-related discrepancies. In AC-1, an anomalous rps19 feature spanning the entire plastome was excluded from the gene count, as it represented an annotation artifact in addition to the correctly located rps19 feature. After correction, AC-1 contained 86 CDS copies compared with 85 in AC-2; the remaining difference was associated with the annotation of psbL, which was annotated as a 117-bp CDS in AC-1 but not in AC-2. Similarly, differences between AA-1 and AA-2 involved the annotation of ccsA and trnL-UAA, respectively. In AA-2, sequence inspection identified a highly similar sequence corresponding to trnL-UAA, indicating that its absence from the annotation does not necessarily represent gene loss.

Analysis of the junctions between the LSC, SSC, and IR regions showed a virtually identical architecture among the plastomes (**Figure 2**). The genes *rps19*, *rpl22*, *ycf1*, *ndhF*, *trnN*, *rpl2*, and *trnH* were consistently located in the same junctional regions, with only minor variations observed in distances from the boundaries between regions. The main differences corresponded to slight expansions and contractions of the IR regions, particularly in *A. nummularia* and other species with plastomes close to 152 kb, which exhibited IR regions of approximately 25.2 kb, whereas species with plastomes close to 150 kb showed IR regions of approximately 24.5 kb.

**Figure 2:**
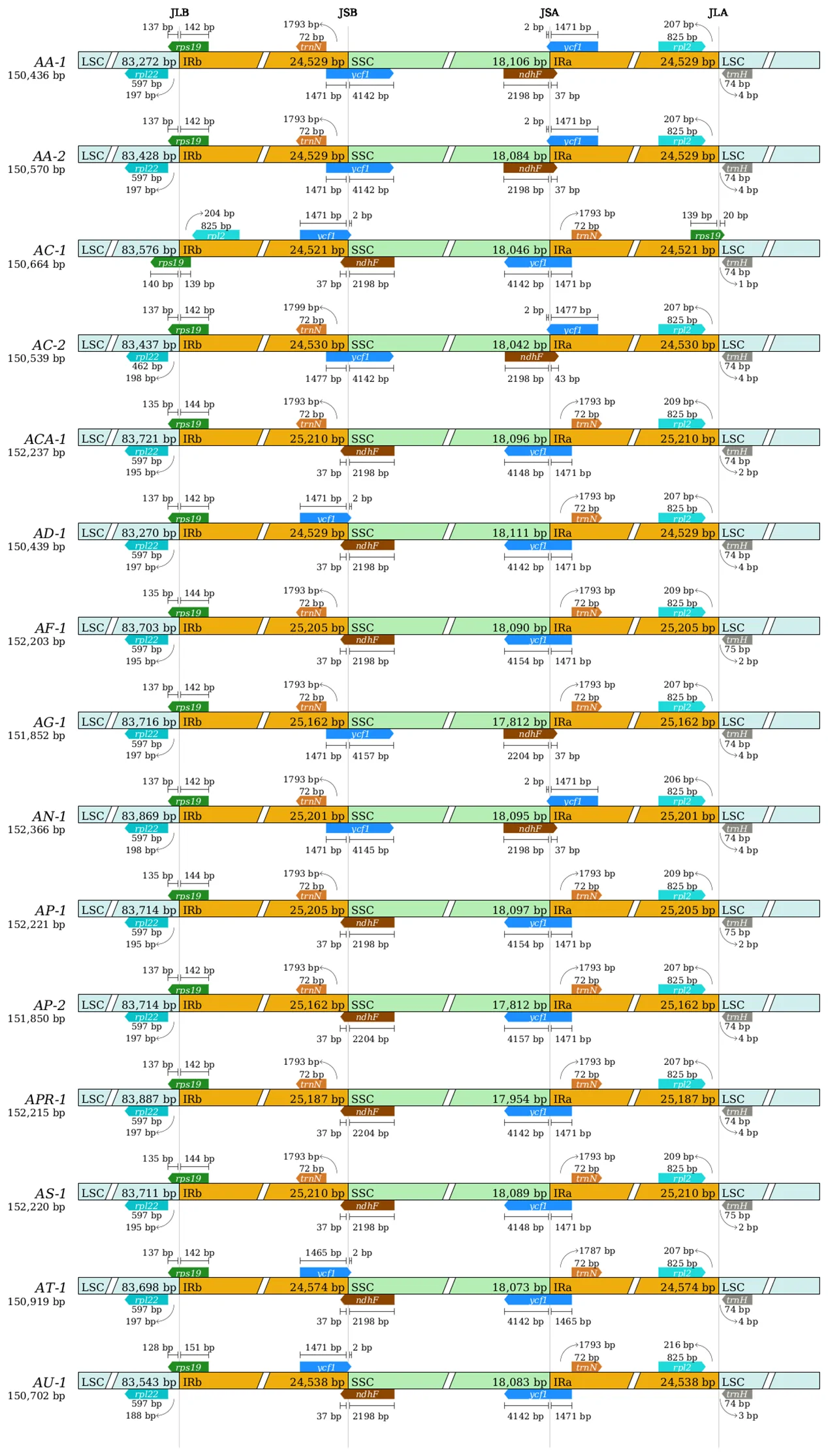
Comparison of the boundaries between LSC, SSC, IRa, and IRb regions in chloroplast genomes of *Atriplex* species. Relative positions of the genes located at the JLB (LSC/IRb), JSB (IRb/SSC), JSA (SSC/IRa), and JLA (IRa/LSC) junctions are shown, along with the distances (bp) between the genes and each boundary. The observed variations reflect slight expansion and contraction events of the IR regions among the compared species.

The chloroplast genomes showed a high degree of sequence conservation, with most coding regions exhibiting sequence identities above 90%, whereas the highest levels of divergence were concentrated in intergenic regions and, to a lesser extent, in a small number of coding genes (**Supplementary Figure S3**). This pattern is consistent with the evolutionary conservation typically observed in angiosperm plastomes, in which coding regions remain highly conserved while non-coding regions accumulate most sequence variation.

Overall, these results indicate that the analyzed Atriplex plastomes are highly conserved in their overall architecture and gene organization, with limited structural variation and localized sequence divergence. The minor differences observed in reported gene numbers are largely attributable to annotation and counting criteria rather than to extensive variation in gene content.

### 3.3 Genomic variation

Comparative analysis of the plastomes identified numerous SNPs and InDels among the analyzed *Atriplex* species (**Figure 3**). The total number of variants differed among comparisons, with more SNPs than InDels observed in all evaluated species.

**Figure 3:**
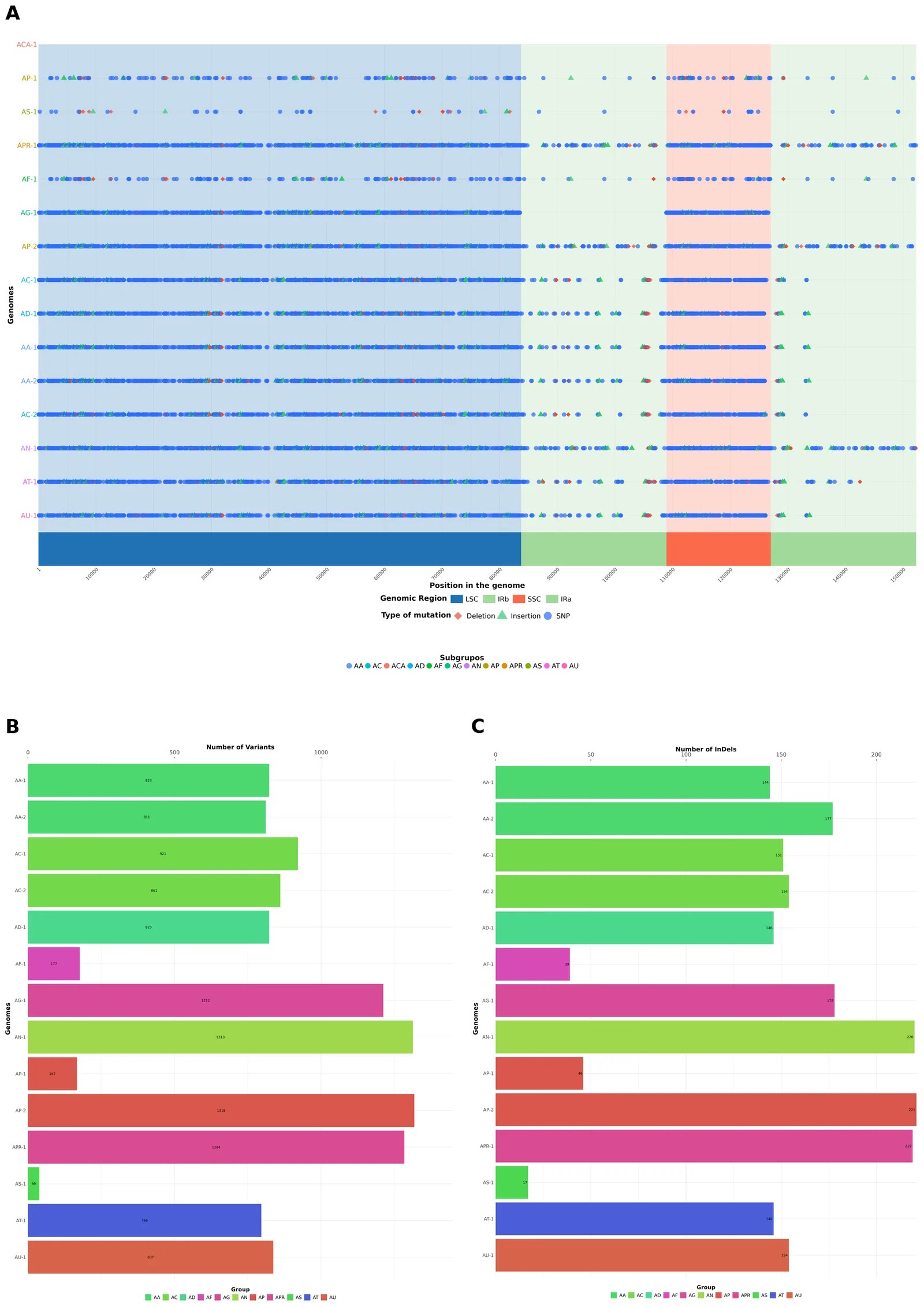
Genomic variation of *Atriplex* species plastomes compared to that of ACA-1. **(A)** Distribution of SNPs and InDels along the chloroplast genome. LSC, SSC, and IR regions are indicated by different background colors. **(B)** Total number of SNPs identified in each species relative to the reference plastome. **(C)** Total number of InDels detected in each comparison. The relative composition of SNP and InDel events is presented in **Supplementary Figure S3**.

The distribution of variants across the genome showed that most SNPs and InDels were concentrated in the LSC region, whereas the IRa and IRb regions showed relatively low variant density. The SSC region showed an intermediate level of variation (**Figures 3B, C**). This distribution was also reflected in the relative abundance of variants, with a similar pattern of base changes observed. Only the *A. sibirica* genome showed a higher proportion of adenine-to-thymine substitutions, at 15.4%, compared with 2%–7% in the other genomes. The proportion of InDels showed a proportional change between insertions and deletions (**Supplementary Figure S4**). The similar distribution pattern observed among species further supports the highly conserved organization of *Atriplex* plastomes despite localized differences in sequence divergence.

Comparison of the Atriplex plastomes using MUMmer4 identified single nucleotide polymorphisms (SNPs) and insertions/deletions (InDels) relative to the A. centralasiatica plastome (ACA-1), which served as the reference sequence. The number of variants therefore represents pairwise divergence from ACA-1 rather than an intrinsic measure of genomic variability within individual species. Among the analyzed plastomes, A. patens (AP-2), A. nummularia (AN-1), A. prostrata (APR-1), and A. gmelinii (AG-1) showed the highest numbers of SNPs relative to ACA-1, whereas A. sibirica (AS-1), A. patens (AP-1), and A. fera (AF-1) showed the lowest numbers. The Chilean plastomes included in the comparison showed intermediate levels of divergence relative to ACA-1, with SNP counts ranging from 796 to 921 among A. taltalensis (AT-1), A. atacamensis (AA-1 and AA-2), A. canescens (AC-1 and AC-2), A. deserticola (AD-1), and A. undulata (AU-1).

The distribution of variants across the plastome was heterogeneous, with SNPs and InDels predominantly located in the LSC region, whereas the IR regions showed comparatively fewer variant positions. The SSC region displayed an intermediate pattern of variation (**Figure 3A**). These results describe sequence differences relative to the selected reference plastome and therefore should not be interpreted as estimates of population-level genetic diversity.

Analysis of the InDels revealed a pattern comparable to that observed for the SNPs, with a relatively high accumulation of events in the LSC region and low frequency within the IR regions. Taken together, these results indicate that plastome variation within the genus *Atriplex* is heterogeneously distributed, concentrating mainly in single-copy regions.

### 3.4 Repetitive sequences

SSRs were identified in all plastomes, with a similar number of repeats observed among the *Atriplex* species (**Figure 4A**). Most SSRs were mononucleotide repeats (1, between 45–56), followed by dinucleotides (2, between 11–7), whereas longer repeats (pentanucleotide [5, only once]- and hexanucleotide [6, between 2–1]) were infrequent.

**Figure 4:**
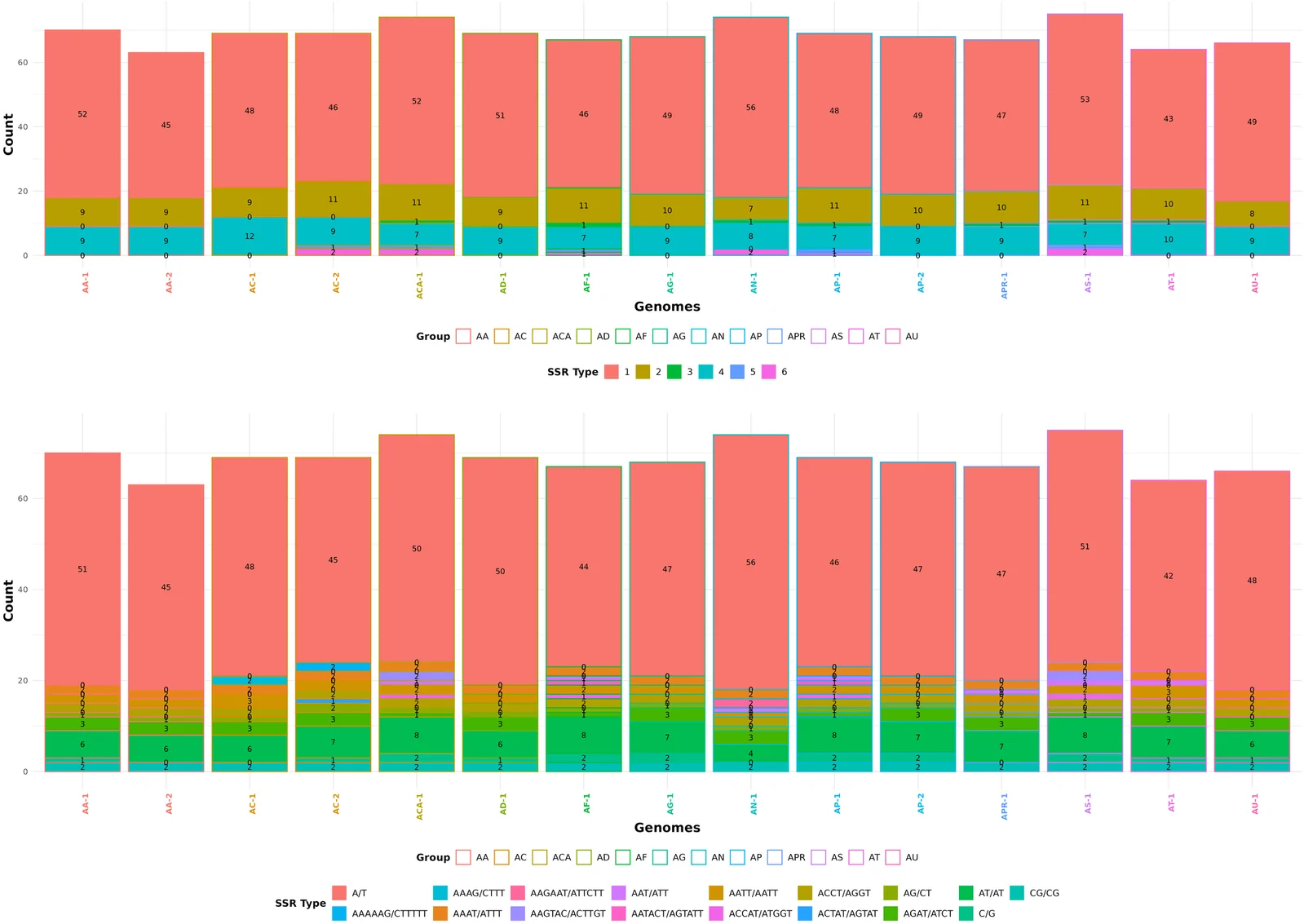
Characterization of SSRs identified in the chloroplast genomes of *Atriplex* species. **(A)** Distribution of the number of SSRs according to the length of the repetitive motif (mono-, di-, tri-, tetra-, penta-, and hexanucleotides). **(B)** Frequency of different SSR motifs detected by MISA in each plastome.

Analysis of repetitive motifs showed a clear predominance of adenine–thymine (A/T)- rich repeats, with 42–56 such repeats identified per plastome, whereas motifs with high G/C content were considerably less frequent (1–2 per plastome) (**Figure 4B**). This pattern was consistent across all analyzed species and coincided with the low overall GC content characteristic of chloroplast genomes. To further characterize their genomic distribution, the 1, 030 SSRs identified across the 15 *Atriplex* plastomes were classified based on their genomic context. A total of 531 SSRs (51.55%) were located in intergenic spacer (IGS) regions, whereas 299 SSRs (29.03%) overlapped annotated coding sequences (CDSs). The remaining 200 SSRs (19.42%) were located within other annotated genic features, mainly non-CDS features such as tRNA and rRNA annotations. Thus, SSRs were more frequently associated with intergenic regions than with protein-coding sequences in the analyzed plastomes (**Supplementary Table S3**). Mononucleotide SSRs predominated in all plastomes, whereas penta- and hexanucleotide repeats were scarce (**Figure 5**; **Supplementary Tables S4**, **S5**).

**Figure 5:**
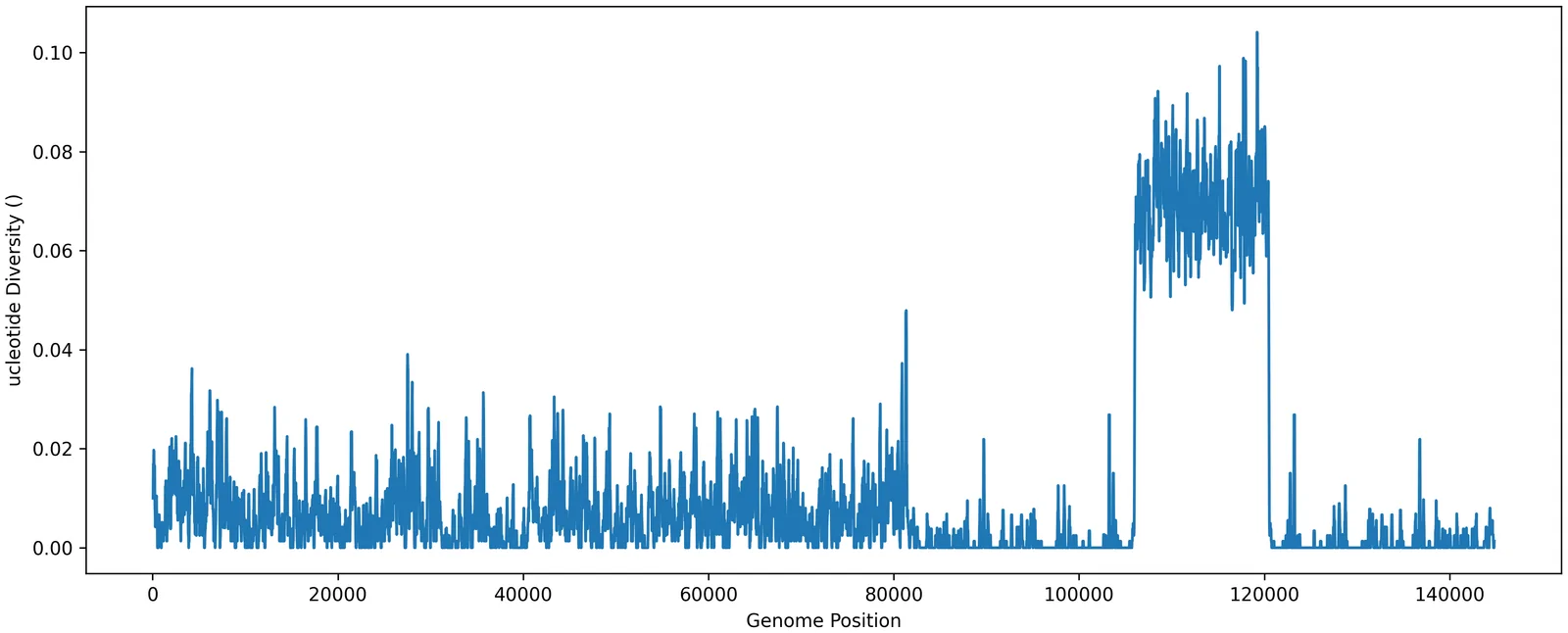
Distribution of Pi along the chloroplast genome of *Atriplex* species, estimated using a sliding window analysis. The x-axis represents genomic position, whereas the y-axis shows Pi values. Peaks correspond to regions with relatively high sequence variability among the analyzed plastomes.

Analysis of long repeats primarily identified forward and palindromic repeats, and reverse and complementary repeats were scarce. Furthermore, most repeats were 20–40 bp long (**Supplementary Figure S5**). The complete distribution of long repeat types and repeat length classes is presented in **Supplementary Figure S5** and **Supplementary Tables S6** and **S7**.

### 3.5 Nucleotide diversity (π) and hypervariable regions

Nucleotide diversity was unevenly distributed across the *Atriplex* plastomes, with most regions showing low levels of variation and only a limited number of localized peaks of increased nucleotide diversity (**Figure 5**).

The highest levels of variation was concentrated in specific plastome regions of the plastome, indicating that sequence variability was not uniformly distributed across the chloroplast genome. These regions coincided with the sectors that previously showed relatively high accumulation of variants in SNP, InDels, and mVISTA analyses, confirming that they constituted the main hotspots of variation within the genus *Atriplex*.

Analysis by coding regions showed that only a small number of genes exhibited above-average Pi values (**Figure 6A**), whereas the majority remained highly conserved. Similarly, several intergenic regions showed above-average π levels (**Figure 6B**), demonstrating that most plastome variation was concentrated outside the coding sequences.

**Figure 6:**
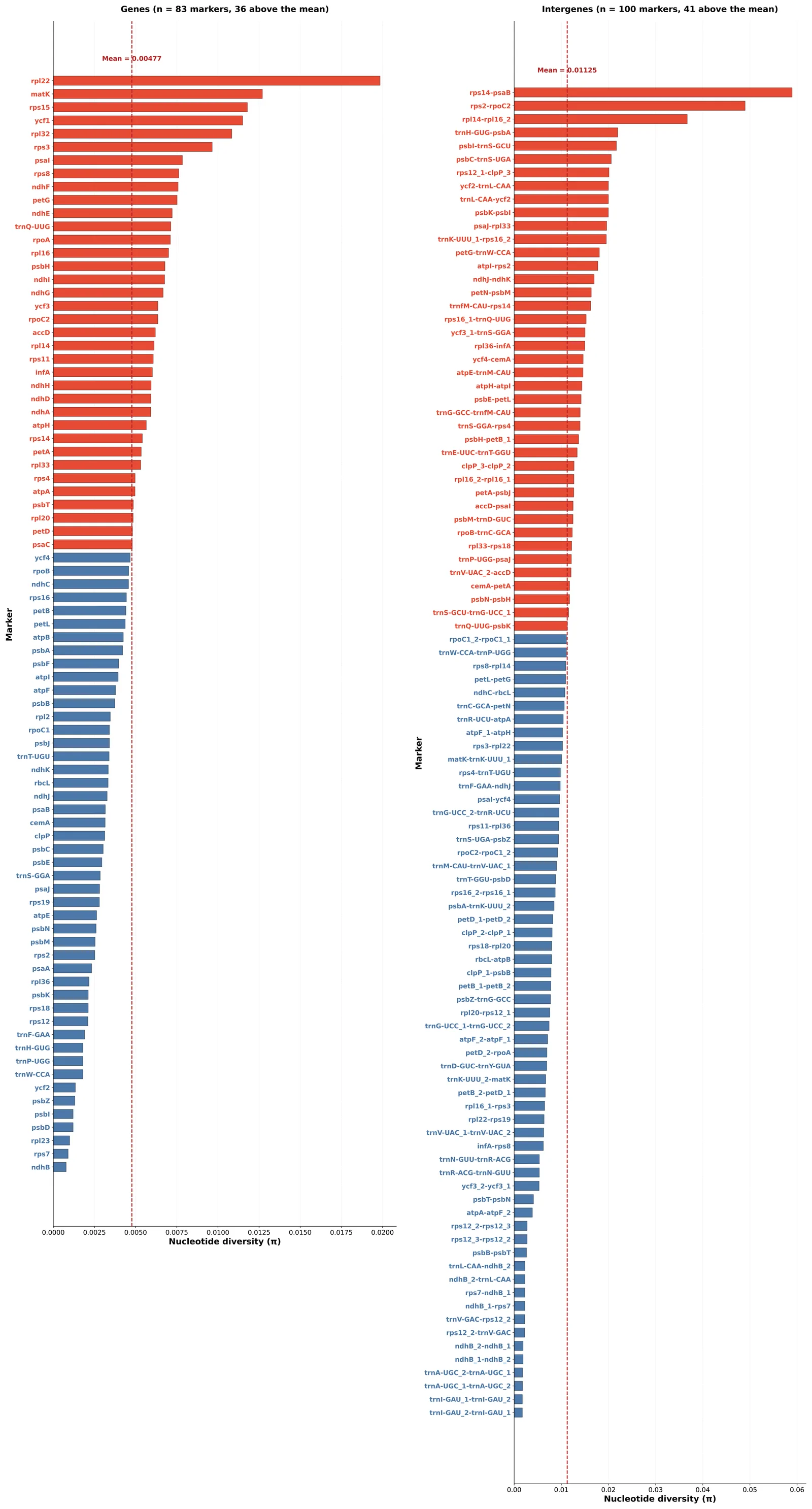
Pi values of the coding and intergenic regions of the *Atriplex* chloroplast genome. **(A)** Ranking of genes according to their Pi values. **(B)** Ranking of intergenic regions ordered from highest to lowest Pi values. The red dotted line indicates the average Pi for each dataset.

Across the 15 *Atriplex* plastomes, the overall nucleotide diversity was π = 0.011751. The alignment contained 9, 720 segregating sites, with Watterson’s θ estimated at 0.020632. Because the dataset represents multiple *Atriplex* species rather than a population sample, these statistics were treated as descriptive measures of sequence variation across the analyzed pastomes and were not interpreted as population-level estimates. Additional descriptive statistics, including haplotype diversity, segregating sites, and Tajima’s D, are provided in **Supplementary Table S8** and were not used for demographic or population-genetic inference.

The hypervariable regions identified in this study represent promising candidates for the development of molecular markers and provide valuable phylogenetic signal for comparative, phylogeographic, and conservation genetic studies within the genus *Atriplex*.

To further evaluate the phylogenetic signal of the hypervariable regions, maximum-likelihood trees were reconstructed for selected individual loci and concatenated datasets and compared with the complete-plastome topology. The *ycf1* tree recovered all 12 internal splits observed in the complete-plastome tree, corresponding to 100% topological concordance. The rpoC2 and matK trees recovered 10 of 12 splits (83.33%), whereas rpl32 and rps15 recovered 4 and 6 splits, respectively. Concatenation of the hypervariable genes recovered 11 of the 12 splits (91.67%), with high mean support among shared splits (97.39% UFBoot and 97.91% SH-aLRT). Similarly, the concatenation of all hypervariable loci recovered 11 of 12 splits (91.67%), with mean support values of 96.95% UFBoot and 96.64% SH-aLRT. The remaining individual and concatenated datasets showed lower levels of topological concordance, indicating that the phylogenetic signal varied among the identified hypervariable regions (**Supplementary Table S9**).

A comparison of the individual reference bipartitions showed that the only split from the complete-plastome topology not recovered by either the concatenated hypervariable genes or the complete hypervariable dataset was the clade comprising AA-1, AA-2, AD-1, AT-1, and AU-1, which showed moderate support in the complete-plastome tree (59.1 UFBoot/61 SH-aLRT). The recovery of each reference bipartition across the individual and concatenated datasets is summarized in **Supplementary Table S10**.

### 3.6 Phylogenetic relationships

Phylogenetic analysis based on complete chloroplast genomes recovered a monophyletic clade corresponding to the sampled *Atriplex* species, clearly separated from the genera used as outgroups (*Beta*, *Chenopodium*, *Dysphania*, *Oxybasis*, and *Limonium*) (**Figure 7**). Most nodes showed maximum statistical support values (SH-aLRT = 100; UFBoot = 100), demonstrating robustness of the phylogenetic reconstruction.

**Figure 7:**
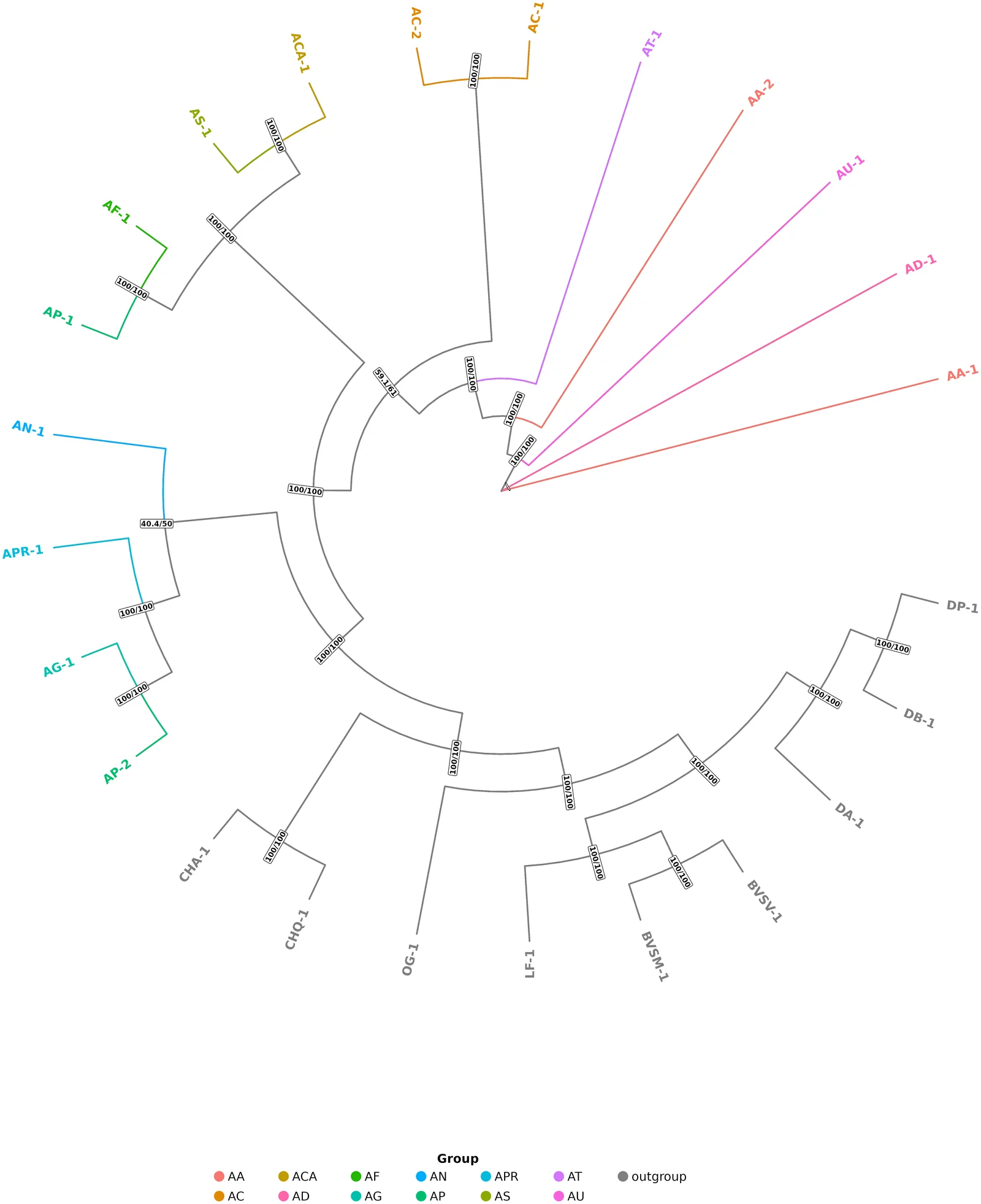
Maximum likelihood phylogenetic tree reconstructed from complete chloroplast genomes of species of the genus *Atriplex*. The tree was inferred using IQ-TREE with the evolutionary model selected by ModelTest-NG. The support values corresponding to UFBoot and SH-aLRT are indicated at the nodes. Species belonging to the genera *Beta, Chenopodium, Dysphania, Oxybasis*, and *Limonium* were used as outgroups.

Several well-supported subclades were identified within the genus *Atriplex*. The plastomes of *A. canescens*, *A. centralasiatica*, *A. sibirica*, *A. fera*, *A. gmelinii*, *A. patens*, and *A. prostrata* formed consistent groupings with high support values, whereas those of *A. atacamensis*, *A. deserticola*, *A. taltalensis*, and *A. undulata* occupied independent positions within the main clade of the genus.

The phylogenetic position recovered for *A. taltalensis* was consistent with the comparative genomic analyses. Together, the high structural conservation of the plastomes, the distribution of SNPs and InDels, and the localized patterns of nucleotide diversity indicate that complete chloroplast genomes provide substantial phylogenetic signal for resolving relationships among the analyzed *Atriplex* plastomes.

### 3.7 Selective pressure on plastid genes

Ka/Ks analyses showed that most protein-coding genes exhibited ratios below 1, consistent with strong functional constraint and predominant purifying selection across the analyzed plastomes (**Figure 8**). A limited number of genes, including *ndhA*, *psbH*, *psbM*, *rpl16*, *rpl22*, and *ycf1*, showed elevated Ka/Ks ratios (>1) in specific pairwise comparisons with the reference plastome. These elevated ratios represent localized signals of increased nonsynonymous substitution relative to synonymous substitution and were therefore not interpreted as definitive evidence of positive selection. The distribution of Ka/Ks values across genes is summarized in **Figure 8**, while the complete set of estimates is provided in **Supplementary Table S11**.

**Figure 8:**
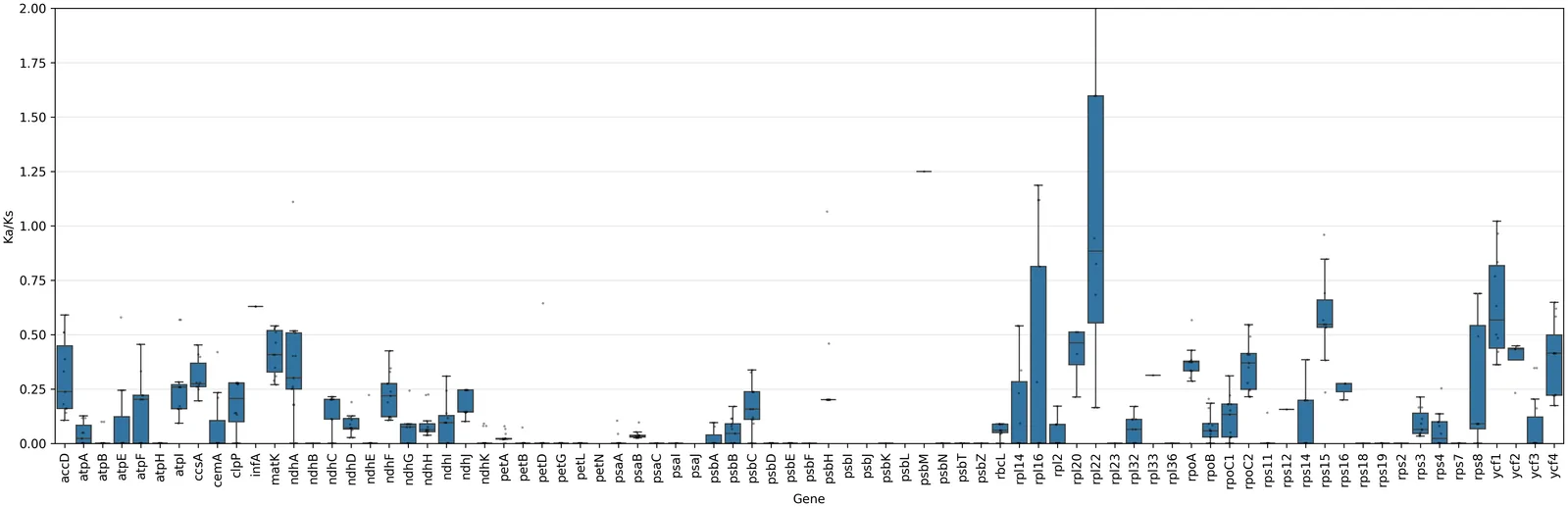
Distribution of Ka/Ks ratios for plastid protein-coding genes in *Atriplex*. Boxplots represent Ka/Ks values obtained from pairwise comparisons between individual plastomes and the selected reference plastome. The center line represents the median, boxes indicate the interquartile range (Q1–Q3), whiskers represent 1.5 times the interquartile range, and dots indicate outliers. Most genes exhibited Ka/Ks ratios below 1, consistent with strong functional constraint across the analyzed plastomes. A limited number of genes showed elevated Ka/Ks ratios (>1) in specific comparisons. Ratios were interpreted as elevated pairwise Ka/Ks signals rather than definitive evidence of positive selection. Comparisons with Ks <0.001 or Ka/Ks >10 were excluded from summary statistics.

## 4 Discussion

### 4.1 Structural conservation of the plastome

The chloroplast genomes of *Atriplex* species showed high conservation in terms of size, gene organization, and GC content. All plastomes exhibited the typical quadripartite structure of angiosperms, comprising an LSC region, an SSC region, and two IR regions, with no evidence of major structural rearrangements.

This pattern coincides with that previously described for *Amaranthaceae* species, in which plastomes exhibit highly conserved gene order and organization of repeated regions (Huang et al., 2024; Maździarz et al., 2025). Similarly, several comparative studies on *Chenopodiaceae sensu lato* have revealed that the evolution of the chloroplast genome occurs mainly through the gradual accumulation of substitutions and InDels, rather than through large chromosomal rearrangements (Yao et al., 2019; Zhang, 2022).

The small differences observed in the length of the IR regions and position of genes located at their boundaries, particularly *rps19*, *ycf1*, *ndhF*, *trnN*, and *rpl2*, correspond to the usual pattern of expansion and contraction of IR regions described in numerous angiosperm plastomes (Wei et al., 2023). Consequently, the structural organization observed in *A. taltalensis* reflects the high evolutionary stability of the plastome within the genus. This structural conservation was also reflacted in codon usage patterns, which showed a high conservation among *Atriplex* species, with a marked preference for codons terminated in adenine or uracil. This pattern has been widely documented in angiosperm plastomes and is primarily attributed to the low GC-content characteristic of chloroplast genomes, particularly at the third codon position (Sharpe et al., 2020; Xu et al., 2024). Furthermore, the similarity observed among species within the genus is consistent with comparative studies indicating that codon usage remains highly conserved in plastomes of closely related lineages, reflecting evolutionary and functional constraints on plastid protein translation (Wicke et al., 2011).

This pattern is consistent with the evolutionary conservation typically observed in angiosperm plastomes, in which coding regions remain highly conserved while non-coding regions accumulate most sequence variation.

### 4.2 Plastome variation

The chloroplast genomes of *Atriplex* were highly conserved in genome structure, gene content, and sequence identity, with variation restricted to a limited number of genomic regions. Structural comparisons revealed only minor differences in IR boundaries, whereas SNP, InDel, and nucleotide diversity analyses consistently identified a small number of evolutionary hotspots concentrated mainly in intergenic regions and a few coding genes. Despite this overall conservation, these localized regions exhibited significantly higher levels of sequence variation than the remainder of the plastome.

Despite the high structural conservation observed among *Atriplex* plastomes, comparative analyses revealed regions with significantly higher-than-average levels of variation. Sequence identity, SNPs, InDels and nucleotide diversity consistently identified a limited number of evolutionary hotspots, concentrated mainly in intergenic regions and a few specific coding genes. The relatively high variability observed in non-coding regions is consistent with the widely documented trend in plants, in which intergenic sequences experience less selective pressure and, therefore, accumulate mutations at a higher rate than coding regions (Campbell and Gowri, 1990; Wang et al., 2022). This behavior explains why a large proportion of SNPs and InDels identified in this study was located outside functional genes.

Furthermore, some genes, such as *ycf1*, *rpoC2*, and *rpl32*, showed high nucleotide diversity. These genes have been previously recognized as highly informative regions for phylogenetic and phylogeographic studies owing to their high rate of evolution within the plastome (Shaw et al., 2007; Zhang et al., 2018). The Ka/Ks analysis showed that most plastid protein-coding genes exhibited ratios below 1, consistent with strong functional constraints and predominant purifying selection across the analyzed plastomes (Dong et al., 2012, Dong et al., 2015). A limited number of genes, including *ycf1*, *rpl16*, *rpl22*, *ndhA*, *psbH*, and *psbM*, exhibited elevated Ka/Ks ratios in specific pairwise comparisons with the reference plastome (Yang et al., 2022b; Zhao et al., 2026). These signals should be interpreted cautiously because elevated pairwise Ka/Ks ratios, particularly when Ks values are very low, do not by themselves constitute evidence of positive selection. In the present study, comparisons with very low Ks values were excluded to reduce the influence of unstable Ka/Ks estimates. Therefore, the observed elevated ratios should be considered candidate signals of differential selective constraint rather than definitive evidence of positive selection. Formal inference of positive selection would require additional codon-based phylogenetic approaches and broader taxon sampling. Functional studies will be required to determine whether these genes contribute to physiological adaptation under environmental stress. Likewise, the recurrent signal detected in *ycf1* is consistent with previous reports describing this gene as one of the fastest-evolving coding regions in angiosperm plastomes (Geng et al., 2026). In contrast, the events detected in *rpl16*, *rpl22*, and other photosystem genes could reflect specific adaptive processes associated with ecological diversification of the genus *Atriplex*, although these results should be confirmed by analyses with a large number of species and complementary evolutionary approaches.

The results obtained indicate that, although the *Atriplex* plastome remains highly conserved at the structural level, it contains specific regions that concentrate much of the evolutionary variation of the genus. In addition to SNPs, InDels, and highly variable loci, repetitive sequences constitute another important source of plastome variation and have been widely investigated as potential markers for population genetic and evolutionary studies.

### 4.3 Repetitive regions

The identified SSRs showed a clear predominance of adenine- and thymine-rich mononucleotide repeats, a pattern widely reported for chloroplast genomes of angiosperms (Scobeyeva et al., 2021; Xiong et al., 2023). The abundance of A/T motifs is directly related to the low overall GC content characteristic of plastid DNA. Likewise, long repeats were dominated by forward and palindromic repeats, whereas reverse and complementary repeats were scarce. This behavior has also been described in various genera of *Amaranthaceae* and associated with recombination mechanisms and structural maintenance of the plastome, rather than with chromosomal rearrangement processes (Lubna Jan et al., 2025).

In addition to their evolutionary relevance, the SSRs identified here represent candidate plastid markers for future population genetic studies. However, their potential utility for assessing genetic diversity and population structure in *A. taltalensis* has not yet been validated. Because the present study includes a single plastome from *A. taltalensis*, these loci should be considered preliminary candidates rather than validated population-level markers. Their polymorphism, transferability, and discriminatory power should be evaluated across multiple individuals and natural populations before their application to population monitoring or to the assessment of gene flow, genetic drift, or inbreeding can be established.

### 4.4 Evolutionary implications

Phylogenetic reconstruction based on whole plastomes recovered well-supported plastome relationships among the analyzed *Atriplex* species. The use of the complete chloroplast genome provides a considerably higher number of informative characters than that achieved using individual plastid markers, increasing phylogenetic resolution in groups with low molecular divergence (Powell et al., 1995; Provan et al., 2001; Singh et al., 2024; Shi et al., 2026).

The comparison of candidate hypervariable regions with the complete-plastome topology further showed that their phylogenetic signal was heterogeneous. In particular, *ycf1* recovered the complete-plastome topology, while the concatenated hypervariable genes and the complete set of hypervariable loci each recovered 91.67% of the internal splits. These results indicate the potential utility of selected hypervariable regions as candidate markers, but also indicate that individual loci may not capture the full phylogenetic signal of the plastome. Therefore, these regions should be considered preliminary candidates for future barcode development rather than validated universal markers for the genus.

Although *A. taltalensis* shares a high degree of structural similarity with other species in the genus, the topology obtained did not recover a clade exclusively comprising the Southamerican species analyzed, suggesting that the current geographical distribution does not necessarily reflect the evolutionary relationships inferred from the plastome. These results are consistent with those of recent studies conducted on other members of Amaranthaceae, in which the use of complete plastomes has significantly increased phylogenetic resolution compared to that achieved by one or a few chloroplast markers (Zhao et al., 2015; Wang et al., 2021; Raiyemo and Tranel, 2023; Kiedaisch et al., 2025).

Although the complete plastome phylogeny recovered well-supported relationships among the analyzed accessions, these relationships should be interpreted specifically as plastome relationships. Chloroplast genomes represent a single, predominantly uniparentally inherited genomic compartment and may therefore capture only part of a species’ evolutionary history (Liu et al., 2026; Shi et al., 2026). This limitation is particularly relevant in *Atriplex*, where hybridization has been reported and chloroplast capture may contribute to discordance between plastid and nuclear evolutionary histories. Consequently, the well-supported plastome topology obtained here should not be interpreted as a complete representation of species-level evolutionary relationships. Future analyses incorporating nuclear genomic data will be important to assess potential cytonuclear discordance and further resolve the evolutionary history of the genus.

Taken together, these findings support the utility of complete plastomes for comparative and systematic studies within the genus *Atriplex*, while highlighting the need for complementary nuclear genomic data to further resolve species-level evolutionary relationships.

### 4.5 Implications for conservation

*A. taltalensis* is an endemic Chilean species restricted to the northern coastal desert, an ecosystem characterized by high endemism and increasing anthropogenic pressure. Despite its ecological importance, genomic information for this species has been virtually nonexistent. The complete chloroplast genome generated in this study therefore represents the first genomic reference available for the species, providing a valuable resource for future studies on population genetics, phylogeography, conservation unit delimitation, and long-term monitoring of natural populations. Furthermore, the availability of this plastome contributes to a broader understanding of the evolution of the genus *Atriplex* in arid environments and establishes a genomic framework for future phylogenomic and conservation-oriented research.

## 5 Conclusions

This study reports the first complete chloroplast genome of *A. taltalensis*, providing a valuable genomic resource for an endangered Chilean endemic species. Comparative analyses demonstrated that, despite the high structural conservation of *Atriplex* plastomes, nucleotide variation is concentrated in a limited number of coding and intergenic regions, highlighting promising candidates for future molecular marker development. Together, these findings establish a solid foundation for future studies on phylogeny, phylogeography, population genetics, and conservation of *Atriplex* species inhabiting arid environments. Future studies integrating nucleotide diversity with variant density analyses and functional validation of candidate genes may further improve the identification of informative markers for conservation and evolutionary research.

## Data Availability

The datasets presented in this study can be found in online repositories. The names of the repository/repositories and accession number(s) can be found below: https://www.ncbi.nlm.nih.gov/genbank/, PRJNA1514774.
