# Supplementary material for: Complete chloroplast genome of Atriplex taltalensis (Amaranthaceae), an endangered endemic species from the Chilean Atacama Desert
Source: Front Plant Sci. 2026 Oct 26;17:1975705. doi: 10.3389/fpls.2026.1975705 (PMC13745725; doi:10.3389/fpls.2026.1975705)
Supplement: Supplementary file 1 [file DataSheet1.docx]

**Supplementary Figure S1. Chloroplast genome maps of the *Atriplex* species analyzed in this study.** Plastome maps are shown for *A. atacamensis* **(A–B)**, *A. canescens* **(C)**, *A. deserticola* **(D)**, *A. nummularia* **(E)**, and *A. undulata* **(F)**. Genes located outside and inside the circle are transcribed clockwise and counterclockwise, respectively. Gene colors indicate functional categories. The inner circle represents GC content across the genome, and the LSC, SSC, and IR regions are indicated for each plastome. The chloroplast genome map of *A. taltalensis* is presented in Figure 1 of the main manuscript.

**A**

**B**

**C**

**D**

**E**

**F**

**Supplementary Figure S2. RSCU patterns of the protein-coding genes in chloroplast genomes of *Atriplex*.** RSCU values are shown for each synonymous codon across all analyzed species. Codons are grouped according to the amino acid they encode, allowing for comparison of codon usage bias among plastomes.

**Figure 3. Comparison of sequence identity of chloroplast genomes from *Atriplex* species using mVISTA with the Shuffle-LAGAN algorithm, with *A. centralasiatica* (ACA-1) as the reference genome.** Coding genes are shown in blue; non-coding regions are presented in pink; and ribosomal RNA genes are shown in light blue. The vertical axis represents the percentage of identity (50%–100%), and horizontal axis represents the position along the plastome. Most coding regions showed high conservation, and the intergenic regions exhibited the highest variability among the analyzed species.

**Supplementary Figure S4. Characterization of sequence variation identified among chloroplast genomes of *Atriplex*. (A)** Relative abundance of different nucleotide-substitution types detected in pairwise genome comparisons. **(B)** Relative proportion of InDel events identified in each plastome. Percentages are calculated considering the total number of sequence variants detected for each genome.

**A**

**B**

**Supplementary Figure S5.** **Characterization of long repeats identified by REPuter in chloroplast genomes of *Atriplex*.** **(A)** Distribution of repeats according to their length. **(B)** Distribution of forward and palindromic repeats detected in each plastome.

**A**

**B**
