# Supplementary material for: Complete chloroplast genome of Atriplex taltalensis (Amaranthaceae), an endangered endemic species from the Chilean Atacama Desert
Source: Front Plant Sci. 2026 Oct 26;17:1975705. doi: 10.3389/fpls.2026.1975705 (PMC13745725; doi:10.3389/fpls.2026.1975705)
Supplement: Supplementary file 2 [file Table1.docx]

**Supplementary Information**

**Supplementary Material S1.** Chloroplast isolation protocol

Chloroplast enrichment was performed using a differential centrifugation protocol as previously described, with slight modifications.^13,14^ All steps were carried out with samples and reagents kept on ice, and centrifugation was performed at 4 °C.

Previously treated leaves were processed by removing main veins and cutting remaining tissue into approximately 1-cm long fragments. Subsequently, the plant material was pre-washed by agitation in 100 mL distilled water per 2 g leaf tissue for 10 min.

The plant material was homogenized using a tissue-to-buffer ratio of 1:3 (w/v), with 50 mL pre-cooled buffer A (0.33 M sorbitol, 50 mM Tris-HCl, pH 7.8, 10 mM ethylenediaminetetraacetic acid [EDTA], 0.1% β-mercaptoethanol, 0.25 M ascorbic acid, and 1%–2% PVP-40 or polyvinylpolypyrrolidone) per 2 g tissue. The material was homogenized using a homogenizer until a uniform suspension was obtained.

The resulting homogenate was filtered twice through four layers of gauze to remove large cell debris. The recovered filtrate was equally divided into 50-mL conical tubes and centrifuged at 500 ×g for 20 min. The resulting supernatant was carefully transferred to clean tubes and subsequently centrifuged at 3,500 ×g for 10 min to pellet down the chloroplasts.

After discarding the supernatant, the green pellet was gently resuspended in 0.5 mL pre-cooled buffer B (0.33 M sorbitol, 50 mM Tris-HCl pH 7.8, 5 mM EDTA, and 1% PVP-40). The suspension was centrifuged at 2,500 ×g for 12 min, and the supernatant was discarded. This washing procedure was repeated once again to improve the purity of the chloroplast fraction.

The final pellet was kept suspended in a wash buffer previously described and stored at 4 °C until further processing.^13^

**Supplementary Material S2.** Chloroplast lysis and DNA extraction protocol

Enriched chloroplasts were recovered by centrifugation at 2,500 ×g for 12 min at 4 °C after prior storage. The supernatant was then discarded, and the pellet was used for chloroplast DNA extraction using a cetyltrimethylammonium bromide (CTAB)-based protocol.

For cell lysis, the pellet was resuspended in 0.5 mL CTAB buffer preheated to 65 °C (100 mM Tris-HCl pH 8.0, 25 mM EDTA, 1.5 M NaCl, and 2% CTAB), to which 2 μL proteinase K (20 mg/mL) and 5 μL fresh β-mercaptoethanol (1% v/v of the total buffer volume) were added. The mixture was homogenized by gentle pipetting and incubated at 65 °C for 60 min, with gentle inversions after every 20 min to promote complete lysis of the chloroplast material.

After incubation, the samples were allowed to cool to ambient temperature and extracted with an equivalent volume of phenol:chloroform (1:1). The mixture was homogenized by gentle inversion and centrifuged at 12,000 ×g for 10 min. The upper aqueous phase was carefully transferred to a new tube.

RNA was removed by adding RNase A (1 mg/mL) at a ratio of 1 μL per 100 μL recovered aqueous phase. Samples were incubated for 15–30 min at 37 °C with constant shaking at 60 rpm.

Subsequently, a second extraction was performed using an equivalent volume of chloroform:isoamyl alcohol (24:1), followed by centrifugation at 12,000 ×g for 10 min. The resulting aqueous phase was carefully recovered for DNA precipitation.

DNA was precipitated by adding 0.7 volume of cold isopropanol (-20 °C), followed by incubation for 20 min at -20 °C. The samples were centrifuged at 12,000 ×g for 20 min at 4 °C, and the supernatant was subsequently discarded.

The DNA pellet was washed with 700 μL cold 70% ethanol and centrifuged again at 12,000 ×g for 20 min at 4 °C. After removing the supernatant, the pellet was allowed to dry at ambient temperature for 5–15 min.

Finally, DNA was resuspended in 25 μL nuclease-free TE buffer and stored at -20 °C until further use.

**Supplementary Material S3.** Primer sequences, and their average temperatures, used for the amplification of plastid and nuclear markers in different *Atriplex* species.

| **Marker** | **Primer forward** | **Melting temperature (°C)** | **Primer reverse** | **Melting temperature (°C)** |
| --- | --- | --- | --- | --- |
| *matK* | 5' GTT GGA AAA TGC GGG TGA CA 3' | 56,3 | 5' TGA GCC AAC GAT CCA ACC AA 3' | 57 |
| *rbcL* | 5' ATG CTA CCA CAT CGA GCC TG 3' | 57,2 | 5' GCG GGC CTT GGA AAG TTT TT 3' | 56,9 |
| *18S* | 5' CAG TAG TCA TAT GCT TGT CTC AAA 3' | 52,7 | 5' GAC TAG GAC GGT ATC TGA TCG T 3' | 55,3 |

**Supplementary Material S4.** MGI adapters used to prepare sequencing libraries.

| **Adaptor** | **Sequence** |
| --- | --- |
| Forward | 5' AAG TCG GAG GCC AAG CGG TCT TAG GAA GAC AA 3' |
| Reverse | 5' AAG TCG GAT CGT AGC CAT GTC GTT CTG TGA GCC AAG GAG TTG 3' |
